# Comparison of Diabetic Nephropathy Markers in Diabetic Patients With Insomnia Before and After Potassium and Magnesium Supplementation: A Randomized Controlled Trial

**DOI:** 10.1002/hsr2.71738

**Published:** 2026-01-18

**Authors:** Sidra Khalid, Shahid Bashir, Riffat Mehboob, Humaira Waseem, Imran Shahid, Abdullah R. Alzahrani, Uzma Malik, Hani Shalabi, Abdulhadi I Bima, Siti Sarah Maidin, Ragdah Hussain Arif, Hussam M Alim

**Affiliations:** ^1^ Institute of Diet and Nutritional Sciences, Faculty of Allied Health Sciences The University of Lahore Pakistan; ^2^ Lahore Medical Research Center Lahore Pakistan; ^3^ University Institute of Food Science and Technology, Faculty of Allied Health Sciences, The University of Lahore Lahore Pakistan; ^4^ Rotogen Biotech United States; ^5^ Department of Quality Enhancement Cell Fatima Jinnah Medical University Lahore Pakistan; ^6^ Center Data Science and Sustainable Technologies INTI International University & Colleges Malaysia; ^7^ Department of Pharmacology and Toxicology, Faculty of Medicine Umm Al‐Qura University Makkah Saudi Arabia; ^8^ Department of Medicine Mayo Hospital Lahore Pakistan; ^9^ Department of Internal Medicine, Faculty of Medicine University of Jeddah Jeddah Saudi Arabia; ^10^ Department of Clinical Biochemistry, Faculty of Medicine King Abdulaziz University Jeddah Saudi Arabia; ^11^ Respiratory Division, Department of Internal Medicine King Abdul Aziz University Jeddah Saudi Arabia; ^12^ Department of Medicine Umm Al‐Qura University Makkah Saudi Arabia

**Keywords:** glycated hemoglobin, insomnia, insomnia severity index, magnesium, potassium, RA factor, serum ALT level, serum AST level, serum creatinine, serum urea level

## Abstract

**Background & Aim:**

Diabetic nephropathy is a common complication in patients with type 2 diabetes, and insomnia may exacerbate renal dysfunction. This study aimed to evaluate the effects of magnesium and potassium supplementation on diabetic nephropathy markers in diabetic patients with insomnia.

**Methods:**

A single‐blinded randomized controlled trial was conducted on 320 diabetic patients. However, only 290 diabetic patients continued the trial after 60 days follow up. Insomnia was defined by using Insomnia Severity Index (ISI). Supplements dosages were prepared in the form of tablets, characterized as T1; placebo, T2; magnesium (Mg), T3; potassium (K), and T4; as magnesium along with potassium. Serum urea level, serum ALT, serum AST, serum creatinine and HbA1c were quantified in blood (serum) employing a quantitative and highly sensitive enzyme‐labelled immunosorbent assay (ELISA), pre‐and post‐trial.

**Results:**

The total of 290 participants; 93 male and 197 female, were having mean HbA1c and BMI as 7.77 ± 2.10 and 31.00 ± 23.68 respectively. A significant association was revealed by analysis among pre‐ and post‐trial serum urea level, serum creatinine, serum ALT level and magnesium supplementation, *p* = 0.000, *p* = 0.000, *p* = 0.003. Moreover, there was significant association between pre and post‐trial serum ALT, AST level, RA factor level and magnesium in combination of potassium supplementation, *p* = 0.000, *p* = 0.004. 0.000.

**Conclusions:**

Study concluded that magnesium, and in combination with potassium supplementation had significant reducing effects on diabetic nephropathy markers and suppression in the RA factor level.

## 1 Introduction

The diabetes frequency is expanding globally with an estimation that that the number of people with diabetes would have increased to 592 million worldwide [1]. Diabetic nephropathy (DN) is a clinical syndrome that involves disturbance of kidney function in individuals with chronic type 1 and type 2 diabetes mellitus. It is widely spreading and resulting in increase in mortality [2]. In the modern world, DN is a significant healthcare challenge. It is a primary cause of end‐stage renal disease and affects up to 50% of people with diabetes [3]. In addition to direct disturbance of sleep, DM also leads to the sleep deprivation by associating with various chronic illness like obstructive sleep apnea or depression [4].

The liver regulates insulin quantities in blood through storing and releasing glycogen, glucose homeostasis, and the synthesis of inflammatory cytokines. These procedures involve common liver enzymes like aspartate aminotransferase (AST) and alanine aminotransferase (ALT). It has been reported that elevated liver enzyme activity may also be a symptom of inflammation, which can interfere with insulin signaling [5]. Despite being currently known to be linked to rheumatoid arthritis, research has found that the rheumatoid arthritis (RA) factor in some cases may be raised in nephropathy as a marker of immune‐mediated injury to the kidneys [6].

Other considerable biomarkers include creatinine and urea which form the basis of the assessment of kidney diseases with emphasis on the nephropathy. Creatinine is a metabolite of muscles and its high levels signify the reduction in glomerular filtration rate of the kidney [7]. Moreover, urea, a nitrogen containing compound formed from the breakdown of proteins also increase in blood in kidney diseases [8]. Altogether, these are a valuable helping means to diagnose and track changes of the kidney dysfunction more accurately and monitor disease progression to guide further management.

Research shows that dietary supplements may help prevent and treat type 2 diabetes. A study has supported the idea that supplementing with zinc and magnesium may help patients with type 2 diabetes manage their glucose [9]. Moreover, several nutrients such as vitamin D, n‐3 PUFAs, and chromium have produced inconsistent results because of inadequate study design and small sample sizes. More well‐designed, high‐quality studies are required to offer insights into the therapeutic research [10]. Magnesium plays a vital role in boosting metabolic pathways in human body [11]. Literature has highlighted that hypomagnesemia occurs in nearly 30% of patients with diabetes. Increased magnesium intake enhances insulin sensitivity and secretion, along with other major bodily functions [12]. Bherwani et al., reported that the frequency of hypomagnesemia in diabetic patients with DN was considerably higher than found in non‐diabetic groups [13]. Clinical magnesium supplementation could therefore be a tactic to enhance the results of diabetes cases [14].

The body also needs potassium as an electrolyte, essential for maintaining normal intracellular fluid levels. Research has shown that potassium‐rich diets may help patients maintain kidney and heart health [15]. Since diabetes is becoming one of the most challenging conditions to treat worldwide, further research is needed to evaluate the efficacy of nutrients and bioelements in managing diabetes and related syndromes [16]. This study aimed to highlight the effect of supplemental magnesium and potassium on DN markers among diabetic patients with insomnia, to reduce disease burden and comorbidities.

## 2 Material and Methods

**Figure 1:**
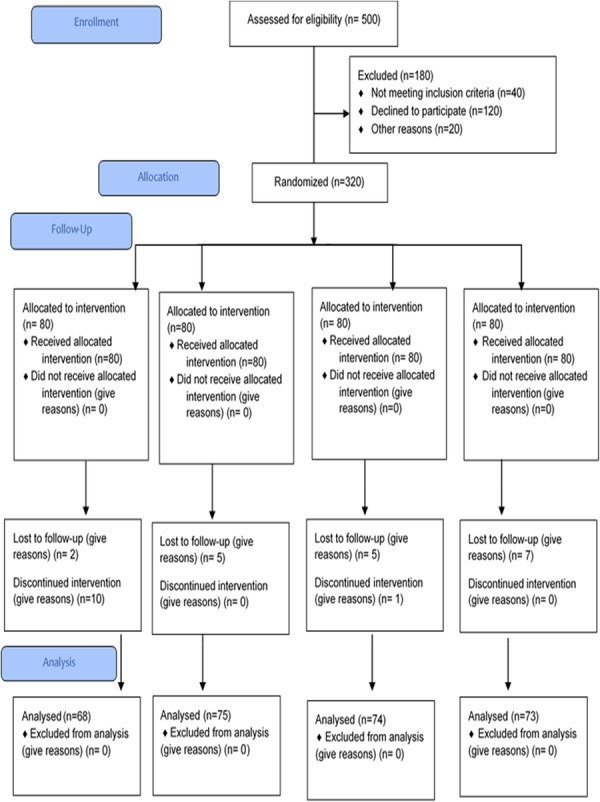
Consort flow diagram.

A single‐blind randomized controlled trial (RCT) was conducted (trial number NCT04642313), and the study flow is illustrated using a CONSORT diagram in Figure 1. RCT was conducted according to the consort flow guidelines. Diabetic nephropathy patients having insomnia as per Insomnia Severity Index (ISI) were selected on the basis of non‐probability purposive sampling. After the ethical approval (IRB‐UOL‐FAHS/760/2020) from the institutional review board of The University of Lahore, Lahore, Pakistan, trial was conducted at the Diabetes Center of Akhuwat Health Services, Lahore. The sample was collected after written informed consent and following ethical guidelines as outlined by declaration of Helsinki.

The sample size was derived by using formula with 95% desired power of the study, 5% level of significance and considering 20% drop out rate. A total of 320 sample size was used with each group (discussed below) containing 80 samples (x4) [17]:

$$n₁=\frac{\left(z₁₋ₐ⁄₂+z₁₋ᵦ\right)²\left(\sigma ₁²+\sigma ₂²⁄r\right)}{\Delta ²}$$

$$r=\frac{n₂}{n₁},\;\Delta =\mu ₁-\mu ₂$$

Z _1 – β_ is the desired power of study= 95%

Z _1‐α/2_ is the desired level of significance= 5%

μ_1_ is the mean in group 1 of insomnia severity (mean of Mg intake group) = 14.14 [17]

μ_2_ is the mean in group 2 of insomnia severity (mean of placebo group) = 15.77 [17]

σ_1_ is standard deviation (SD) in group 1 of insomnia severity (SD of Mg intake group) = 2.68 [17]

σ_2_ is standard deviation (SD) in group 2 of insomnia severity (SD of placebo group) = 1.92 [17]

Ratio (r)= 1

Alpha (α)= 0.05

Beta (β)= 0.2

n (expected sample size) = 66 in each group (x4)

Sample size was adjusted according to 20% drop out rate, with 20% dropout expected sample was 80 (x4) in each treatment group, as there were 4 treatment groups so, total sample size was 320.

The patients were only included when aged between 19 and 65 years old and Diabetic Nephropathy was also diagnosed and when they had a pre‐existing Insomnia as measured by Insomnia Severity Index (ISI) besides receiving a stable Diabetes treatment regimen within the last 3 months before onboarding. All participants continued their prescribed diabetes medications throughout the trial, and no changes in class or dose were allowed.

Patients with co‐morbidities such as cardiovascular disease, severe hepatic insufficiency, non‐diabetic renal disorders, psychiatric disorders, chronic stress, or other sleep disorders (sleep apnea, restless leg syndrome) were excluded to avoid confounding effects on study outcomes. Patients who are known to be influenced positively or negatively by the drugs or who were taking medications affecting sleep, magnesium or potassium metabolism, or renal function were also excluded, as well as in case of imbalanced electrolytes or renal variables. Also, those patients who were on hormonal therapy, alcohol users or those whose substance abuse history had been confirmed were excluded. Pregnant women or women who were already lactating, and women who were unable to meet the demands of the study were also not allowed into the study. All subjects maintained their pre‐study diabetes medication regimen throughout the trial to minimize medication‐related bias. Helsinki guidelines were followed during the trial, and written consent for study participation was obtained from all diabetic patients.

The treatment tablets were manufactured at Paragon Laboratories and were subjected to the approval of FORM‐6 as per the Drug Regulatory Authority, Pakistan under section of Pakistan act of 1976. For treatment group T1 placebo tablets containing starch, for treatment group T2 magnesium tablets containing magnesium gluconate, for treatment group T3 potassium tablets containing potassium chloride and for treatment group T4 magnesium along with potassium tablets containing magnesium gluconate plus potassium chloride were prepared, for trial. Tablets were packed in child‐safe bottle packaging under room temperature. The trial involved controlling of diet, physical activity, and medication use. A standardized diet using Pakistani dietary guidelines was given to the participants with intake of potassium and magnesium limited to less than 75% of the recommended intake. They were asked to continue with their normal daily routine and not to embark on new workout programs.

Study participants were randomly enrolled in treatment groups. Blood samples (pre‐ and post‐trial) were taken by a certified phlebotomist in fasting condition, for glycated hemoglobin, serum ALT, serum AST, serum urea, serum creatinine and serum rheumatoid arthritis (RA) factor. Demographic factors such as age, gender, body mass index (BMI) was noted. Also, as baseline parameters of the study participants, glycated hemoglobin (HbA1c) was measured and insomnia categories according to ISI scale, were defined. Serum urea, serum ALT, serum AST, serum creatinine, and HbA1c were estimated from serum using a quantitative and sensitive enzyme labelled immunosorbent assay (ELISA). Statistical Package for Social Sciences (SPSS) version 25.0 was used to perform statistical analysis on the data. DN markers and age were given as mean ± SD. Consideration was made for *p*‐value ≤ 0.05 significant when conducting the Chi‐square test.

## 3 Results

The mean age of study participants was 49.08 ± 9.51 years (Table 1). The study included 197 (67.9%) female and 93 (32.1%) male participants. The mean HbA1c and BMI were 7.77 ± 2.10% and 31.00 ± 23.68 kg/m², respectively. According to ISI categories, 60% of participants were clinically insomniac.

**Table 1:** Demographic and baseline clinical parameter of study participants.

Demographic and baseline clinical parameter of study participants	
Age (Yrs)	Mean ± SD
	49.076 ± 9.505
HbA1c	7.77 ± 2.10
BMI	31.00 ± 23.68
Gender	
Male	93 (32.07)
Female	197 (67.93)
ISI Categories	
No clinically significant insomnia	6 (2.1%)
Sub‐threshold insomnia	44 (15.2%)
Clinical insomnia (moderate severity)	174 (60.0%)
Clinical insomnia	66 (22.8%)

### 3.1 Serum Urea Levels

Table 2 shows the within‐group comparison of serum urea levels before and after the trial. A paired t‐test revealed no statistically significant difference in T1, T3, and T4 groups (*p *= 0.416, 0.190, 0.491, respectively), whereas T2 (magnesium) showed a significant reduction in serum urea levels (*p* < 0.001, mean difference = 0.37 mg/dL, 95% CI: 0.20–0.54) (Table 2).

**Table 2:** Comparison of serum urea level before and after the trial within treatment groups of study participants.

Serum urea level (mg/dL)			
Treatment groups	*N*	Mean ± SD	*p* value
T1	Pre	33.34 ± 11.19	0.416
	Post	32.03 ± 10.86	
T2	Pre	35.25 ± 8.96	0.000
	Post	34.88 ± 9.47	
T3	Pre	35.77 ± 9.76	0.190
	Post	33.43 ± 11.63	
T4	Pre	31.31 ± 11.75	0.491
	Post	32.81 ± 15.11	

*Note:* T1, Placebo (250mg‐twice a day); T2, Mg (250mg‐twice a day); T3, K (250mg‐twice a day); T4, Mg + K (250mg‐twice a day).

Between‐group analysis (ANOVA) for post‐trial serum urea (Table 3) indicated no statistically significant difference among the four treatment groups (*p* = 0.548, η² = 0.01). Although the Mg group showed significant within‐group change, the Mg‐K combination group (T4) post‐trial mean (32.81 mg/dL) was closest to the placebo.

**Table 3:** Comparison of post‐trial serum urea level of participants among treatment groups.

Post‐trial urea (mg/dL)			
Groups	*N*	Mean ± SD	*p* value
T1	68	32.03 ± 10.86	0.548
T2	75	34.88 ± 9.47	
T3	74	33.43 ± 11.63	
T4	73	32.81 ± 15.11	
Total	290	33.18 ± 11.77	

### 3.2 Serum Creatinine Levels

Within‐group comparisons (paired t‐test, Table 4) demonstrated a significant decrease in T2 (*p* = 0.001, mean difference = 0.19 mg/dL, 95% CI: 0.10–0.28) and a non‐significant decrease in T1, T3, and T4 (*p* = 0.189, 0.105, 0.152).

**Table 4:** Pre‐trial and post‐trial comparison of serum creatinine level within treatment groups.

Serum creatinine level (mg/dL)			
Treatment groups	*N*	Mean ± SD	*p* value
T1	Pre	1.07 ± 0.36	0.189
	Post	1.00 ± 0.27	
T2	Pre	1.13 ± 0.39	0.001
	Post	0.94 ± 0.23	
T3	Pre	1.05 ± 0.36	0.105
	Post	0.96 ± 0.26	
T4	Pre	1.39 ± 2.80	0.152
	Post	0.89 ± 0.62	

*Note:* T1, Placebo (250 mg‐twice a day); T2, Mg (250 mg‐twice a day); T3, K (250 mg‐twice a day); T4, Mg + K (250 mg‐twice a day).

Post‐trial between‐group analysis (ANOVA, Table 5) revealed a significant effect of interventions on creatinine levels (*p* < 0.001, η² = 0.12), with the greatest reduction observed in T4 (from 1.00 to 0.89 mg/dL).

**Table 5:** Post‐trial creatinine comparison among treatment groups of study participants.

Post‐trial creatinine (mg/dL)			
Groups	*N*	Mean ± SD	*p* value
T1	68	1.00 ± 0.27	0.000
T2	75	0.94 ± 0.23	
T3	74	0.96 ± 0.26	
T4	73	0.89 ± 0.62	
Total	290	0.95 ± 0.35	

*Note:* T1, Placebo (250 mg‐twice a day); T2, Mg (250 mg‐twice a day); T3, K (250 mg‐twice a day); T4, Mg + K (250 mg‐twice a day).

### 3.3 Serum ALT Levels

Within‐group paired t‐tests (Table 6) showed significant reductions in T2 (*p* = 0.003, mean difference = 4.1 mg/dL, 95% CI: 1.5–6.7) and T4 (*p* < 0.001, mean difference = 3.6 mg/dL, 95% CI: 1.4–5.8), whereas T1 and T3 were non‐significant (*p* = 0.990, 0.092). Between‐group comparison (ANOVA) for post‐trial ALT levels was not statistically significant (*p* = 0.124, η² = 0.02).

**Table 6:** Pre‐trial and post‐trial comparison of serum ALT levels (mg/dL) within treatment groups of study participants.

Serum ALT Levels (mg/dL)			
Treatment groups	*N*	Mean ± SD	*p* value
T1	Pre	35.2 ± 13.1	0.99
	Post	35.1 ± 17.6	
T2	Pre	38 ± 10.0	0.003*
	Post	33.9 ± 7.0	
T3	Pre	35 ± 11.0	0.092
	Post	31.4 ± 18.0	
T4	Pre	35 ± 10.0	0.000**
	Post	31.4 ± 11.1	

*Note:* T1, Placebo (250 mg‐twice a day); T2, Mg (250 mg‐twice a day); T3, K (250 mg‐twice a day); T4, Mg + K (250 mg‐twice a day).

### 3.4 Serum AST Levels

Within‐group analysis (paired t‐test, Table 7) indicated a significant reduction only in T4 (*p* = 0.004, mean difference = 2.5 mg/dL, 95% CI: 0.8–4.2). No significant changes were observed in T1–T3 (*p* = 0.989, 0.375, 0.071). Post‐trial between‐group comparison was not significant (*p* = 0.087, η² = 0.03).

**Table 7:** Comparison of pre‐ and post‐trial serum AST levels (mg/dL) within treatment groups of study participants.

Serum AST level (mg/dL)			
Treatment groups	*N*	Mean ± SD	*p* value
T1	Pre	32.7 ± 14.5	0.989
	Post	32.56 ± 7.69	
T2	Pre	32.0 ± 7.9	0.375
	Post	31.0 ± 12.0	
T3	Pre	30.7 ± 10.4	0.071
	Post	28.0 ± 8.2	
T4	Pre	30.6 ± 10.6	0.004**
	Post	28.1 ± 8.0	

*Note:* T1, Placebo (250 mg‐twice a day); T2, Mg (250 mg‐twice a day); T3, K (250 mg‐twice a day); T4, Mg + K (250 mg‐twice a day).

### 3.5 RA Factor Levels

Within‐group paired t‐tests (Table 8) showed significant reduction in T4 (*p* < 0.001, mean difference = 8.79 IU/mL, 95% CI: 5.4–12.2), whereas T1–T3 showed no significant changes (*p* = 0.732, 0.561, 0.146). Post‐trial between‐group ANOVA (Table 9) indicated a statistically significant difference among treatment groups (*p* = 0.007, η² = 0.05).

**Table 8:** Comparison of pre‐ and post‐trial RA Factor level (IU/mL) within treatment groups of study participants.

RA Factor level (IU/mL)			
Treatment groups	*N*	Mean ± SD	*p* value
T1	Pre	34.58 ± 11.29	0.732
	Post	34.07 ± 12.31	
T2	Pre	38.12 ± 11.75	0.561
	Post	36.98 ± 11.25	
T3	Pre	37.62 ± 13.46	0.146
	Post	34.70 ± 11.92	
T4	Pre	37.42 ± 10.50	0.000
	Post	28.63 ± 9.35	

*Note:* T1, Placebo (250mg‐twice a day); T2, Mg (250mg‐twice a day); T3, K (250mg‐twice a day); T4, Mg + K (250mg‐twice a day).

**Table 9:** Comparison of post‐trial RA Factor level (IU/mL) among treatment groups in study participants.

Post‐trial RA factor level (IU/mL)			
Groups	*N*	Mean ± SD	*p* value
T1	68	34.07 ± 12.31	0.007
T2	75	36.62 ± 11.25	
T3	74	34.70 ± 11.92	
T4	73	28.63 ± 9.35	
Total	290	33.51 ± 11.21	

*Note:* T1, Placebo (250 mg‐twice a day); T2, Mg (250 mg‐twice a day); T3, K (250 mg‐twice a day); T4, Mg + K (250 mg‐twice a day).

### 3.6 Gender‐Wise Comparisons

Most post‐intervention renal and liver markers did not differ significantly by gender (*p* > 0.05). Post‐ALT levels were slightly higher in males (36.57 ± 12.37 U/L) than females (33.46 ± 10.35 U/L, *p* = 0.026, 95% CI: 0.38–5.84), suggesting a weak gender effect (Table 10).

**Table 10:** Comparison of post‐intervention diabetic nephropathy markers.

Variable	Male (Mean ± SD)	Female (Mean ± SD)	t (df)	*p* value	95% CI (Lower–upper)
Post‐urea (mg/dL)	34.98 ± 12.76	32.64 ± 11.47	1.566 (288)	0.118	–0.60 to 5.29
Post‐creatinine (mg/dL)	1.06 ± 0.49	1.03 ± 0.44	0.370 (288)	0.712	–0.09 to 0.13
Post‐ALT (U/L)	36.57 ± 12.37	33.46 ± 10.35	2.239 (288)	0.026	0.38 to 5.84
Post‐AST (U/L)	31.54 ± 9.10	31.15 ± 9.07	0.342 (288)	0.733	–1.86 to 2.64
Post RA‐factor (IU/mL)	5.10 ± 5.12	5.36 ± 4.89	–0.403 (288)	0.687	–1.48 to 0.98

## 4 Discussion

This study demonstrated that supplementation with magnesium (T2), potassium (T3), and their combination (T4) significantly improved diabetic nephropathy markers, including serum urea, creatinine, ALT, AST, and RA factor, compared with placebo (T1). A randomized controlled trial was carried out in this investigation to compare the impact on diabetic nephropathy markers of either potassium or magnesium in insomniac diabetes patients. The results highlighted the effectiveness of these supplements for altering serum urea, creatinine, ALT, AST, and RA factors in the numerous treatment groups. Among the study participants, 60% were clinically insomniac, building a strong relation between insomnia and diabetic nephropathy; this conforms with a study in which short sleepers (5–6 h) had elevated urea and creatinine levels [18].

Magnesium may improve renal function by enhancing glomerular filtration and reducing nitrogenous waste accumulation, while potassium supports electrolyte balance and vascular health; together they may synergistically reduce renal and liver stress. Clinically, these supplements could serve as adjunct therapy to improve diabetic nephropathy markers and inflammation in insomniac patients

Serum urea and creatinine levels are two pivotal biomarkers screened for diabetic nephropathy [19]. Current study showed that supplementing magnesium reduced the serum urea level in type 2 diabetic nephropathy patients. The evaluation of melatonin intervention versus placebo group of stress‐induced hyperglycemic patients showed insignificant differences in urea and creatinine levels; urea decreased whereas creatinine increase [20]. Moreover, in another research on reducing complications in diabetic rats, melatonin increased urea and creatinine levels in comparison with healthy controls [21]. But in this study, creatinine levels for Mg and Mg‐K groups were significantly than that for placebo group post‐trial. Magnesium could have improved renal function or directly diminished the nitrogenous by‐products in blood. The findings are agreed by the results of Matsuzaki et al., where better renal function was indicated by improved creatinine and urea clearance from serum, which were linked to increased magnesium consumption [22].

Recent studies further support these findings. For example, a cross‐sectional study in Bangladesh reported a high prevalence of hypomagnesaemia among diabetic patients with chronic kidney disease, showing a significant correlation between magnesium levels and renal function [23]. Another randomized single‐blinded clinical trial in Lahore compared magnesium, potassium, and their combination on cholesterol and quality of life in type 2 diabetes patients, demonstrating significant improvements in metabolic markers and well‐being with supplementation [24]. Additionally, a prospective randomized trial in Egypt showed that oral magnesium citrate supplementation improved microalbuminuria, lipid profile, glycemic control, and quality of life in diabetic nephropathy patients, confirming the renoprotective role of magnesium [25]. Together, these latest studies corroborate the beneficial effects of magnesium, and magnesium–potassium co‐supplementation, on renal and metabolic outcomes in diabetes.

Serum creatinine was decreased in all treatments. However, a significant reduction was found magnesium (T2) group. According to literature, in hypertensive rat models, blood creatinine levels were considerably lowered by both potassium and magnesium. It seemed that administering these electrolytes together had a more significant impact. Whereas, treatment of diabetic nephropathy patients with ingestible melatonin tablets, increased blood urea and decreased creatinine [26]. The results pointed the potential benefits of co‐supplementation of potassium and magnesium in controlling creatinine levels, especially in hypertensive disorders. Further research is needed as a higher risk of hypertension is closely linked to insomnia, especially if it is persistent and accompanied by short sleep duration [27].

Liver damage and metabolic diseases, such as diabetes, are frequently linked to elevated ALT and AST levels. Data on liver functions and their markers demonstrated a significant decrease in ALT levels in groups that received magnesium (T2) and magnesium‐potassium (T4). Contrarily, melatonin treatment that promised to reduce stress‐induced hyperglycemia and insulin resistance greatly increased ALT and AST levels, further worsening the condition if given to diabetic nephropathy patients [20]. This study demonstrated that Mg and K supplementation lowered post‐trial AST and ALT concentrations of all three groups to that below than placebo group's concentrations. Whereas, in diabetic rats, melatonin reduced AST and ALT levels, but it could not normalize them to healthy rats' levels [21]. The efficiency of magnesium supplementation on liver enzymes in patients with nonalcoholic fatty liver disease and its role in improving sleep quality in insomniacs have previously been reported [22, 26, 28]. A decrease in ALT may be related to the alteration of metabolic pathways following magnesium intake. Our study is supported by the findings which indicated that potassium salt supplementation demonstrated dramatically lower ALT levels. Potassium may indirectly promote liver health and lower liver enzyme levels by lowering triacylglycerol (TG) levels and atherosclerotic plaques [29]. These results in human investigations of combined magnesium and potassium supplementation have been validated by this current study, suggesting that it may be used as a therapeutic agent to promote liver health that improves insulin signaling. It was observed that the combination treatment group (T4) exercised a decrement effect on AST levels. Combined magnesium‐potassium may reduce liver stress, probably due to a differential effect on cellular metabolism as compared to magnesium supplementation alone.

The prevalence of rheumatoid arthritis is closely associated with diabetes as elevated levels of insulin are found in RA patients and may induce nephropathy due to immune‐mediated kidney damage [30]. This study found that, in addition to placebo, both magnesium‐potassium supplements helped in reducing the RA factor levels. According to one study, dietary magnesium consumption between 181 and 446 mg/day was associated with the lowest incidence of RA. Even after controlling for other variables, a different study discovered a negative relationship between RA and dietary magnesium [31]. Additionally, potassium levels are frequently lower in RA patients than in healthy individuals. RA may benefit from a higher potassium intake either through diet or supplements. To the best of our knowledge, no study has yet determined the combined effect of Mg‐K supplementation on RA factor in diabetic nephropathy patients.

This study was limited by a relatively short intervention period and single‐center design. Future research should evaluate long‐term effects, optimal dosing, and molecular mechanisms of magnesium and potassium supplementation. Additionally, larger multicenter trials are recommended to confirm these findings across diverse populations and explore interactions with other comorbidities and medications.

## 5 Conclusion

The current study demonstrates that magnesium and potassium supplementation, alone or in combination, significantly improves diabetic nephropathy markers, including serum urea, creatinine, and RA factor, in diabetic patients with insomnia. These supplements also support renal and liver function without adding medication burden. Further studies are needed to determine optimal dosages and clarify the molecular mechanisms underlying these benefits.

## Author Contributions

Conceptualization: Sidra Khalid and Shahid Bashir. Data curation: Sidra Khalid, Shahid Bashir, Riffat Mehboob, Humaira Waseem, Imran Shahid, Abdullah R. Zahrani, Hani Shalabi, and Ragdah Hussain Arif. Formal analysis: Siti Sarah Maidin, Sidra Khalid, Shahid Bashir, Riffat Mehboob, and Humaira Waseem. Investigation: Imran Shahid, Abdullah R. Zahrani, Hani Shalabi, Ragdah Hussain Arif, and Hussam M Alim. Methodology: Riffat Mehboob, Humaira Waseem, Imran Shahid, Abdullah R. Zahrani, Hani Shalabi, and Ragdah Hussain Arif. Project administration: Riffat Mehboob, Sidra Khalid, and Shahid Bashir. Supervision: Shahid Bashir. Validation: Siti Sarah Maidin, Sidra Khalid, Riffat Mehboob, and Hussam M. Alim. Visualization: Riffat Mehboob and Humaira Waseem. Writing ‐ original draft: Sidra Khalid, Shahid Bashir, Riffat Mehboob, Humaira Waseem, and Imran Shahid. Writing ‐ review & editing: Abdullah R. Zahrani, Hani Shalabi, Ragdah Hussain Arif, and Hussam M. Alim. All the authors have edited and approved the final version of the manuscript.

## Ethics Statement

All authors have read and approved the final version of the manuscript. Sidra Khalid, corresponding author, had full access to all of the data and takes complete responsibility for the integrity of the data and the accuracy of the data analysis.

## Conflicts of Interest

The authors declare no conflicts of interest.

## 1 Transparency Statement

The Sidra Khalid affirms that this manuscript is an honest, accurate, and transparent account of the study being reported; that no important aspects of the study have been omitted; and that any discrepancies from the study as planned have been explained.

## Data Availability

The data will be available upon reasonable request from the corresponding author.
